# Pandemic Influenza A(H1N1)pdm09 Seroprevalence in Sweden before and after the Pandemic and the Vaccination Campaign in 2009

**DOI:** 10.1371/journal.pone.0053511

**Published:** 2012-12-28

**Authors:** Andreas Mörner, Andreas Bråve, Anna-Maria Kling, Sharon Kühlmann-Berenzon, Katarina Krook, Mona Hedenskog, Irene Silhammar, Margaretha Ljungman, Åke Örtqvist, Sören Andersson, Maria Brytting, Rigmor Thorstensson, Annika Linde

**Affiliations:** 1 Department of Diagnostics and Vaccinology, Swedish Institute for Communicable Disease Control, Solna, Sweden; 2 Department of Preparedness, Swedish Institute for Communicable Disease Control, Solna, Sweden; 3 Department of Analysis and Prevention, Swedish Institute for Communicable Disease Control, Solna, Sweden; 4 Department of Communicable Diseases Control and Prevention, Stockholm County, Sweden; 5 Department of Medicine, Unit of Infectious Diseases, Karolinska Institutet, Karolinska Solna, Stockholm, Sweden; University of Hong Kong, Hong Kong

## Abstract

The immunity to pandemic influenza A(H1N1)pdm09 in Sweden before and after the outbreaks in 2009 and 2010 was investigated in a seroepidemiological study. Serum samples were collected at four time points: during 2007 (n = 1968), in October 2009 (n = 2218), in May 2010 (n = 2638) and in May 2011 (n = 2513) and were tested for hemagglutination inhibition (HI) antibodies. In 2007, 4.9% of the population had pre-existing HI titres ≥40, with the highest prevalence (20.0%) in 15–24 year-olds, followed by ≥80 year-olds (9.3%). The overall prevalence of HI titres ≥40 had not changed significantly in October 2009. In May 2010 the prevalence had increased to 48.6% with the highest percentages in 5–14 year-olds (76.2%) andlowest in 75–79 year-olds (18.3%). One year later the prevalence of HI titres ≥40 had increased further to 52.2%. Children 5–14 years had the highest incidence of infection and vaccine uptake as well as the highest post-pandemic protective antibody levels. In contrast, the elderly had high vaccine uptake and low attack rate but low levels of protective antibodies, underlining that factors other than HI antibodies are involved in protection against influenza A(H1N1)pdm09. However, for all age-groups the seroprevalence was stable or increasing between 2010 and 2011, indicating that both vaccine- and infection-induced antibodies were long-lived.

## Introduction

The first cases of influenza A(H1N1)pdm09 in Sweden were identified in early May 2009, and the infection was included among notifiable communicable diseases on May 15, 2009. Sporadic cases, most of which were travel-related, occurred during the spring and early summer. Two small peaks followed. The first, in mid-July, consisted mainly of imported cases and was largely the result of intense sampling due to contact tracing, which was mandatory until July 16. The second small peak occurred at the end of August, when schools started. The spread was then possibly interrupted by rhinovirus infections [1]. Massive spread of the virus started in mid-October and the epidemic peaked in mid-November. Altogether 11,009 cases (116 per 100,000 population) were laboratory-confirmed during the 2009–2010 season. The previously highest reported number of laboratory-confirmed influenza cases was in the season 2004–2005 with totally 2015 laboratory-confirmed influenza diagnoses. The reported incidence 2009 was highest in children 0–14 years (295/100,000), while very few persons over the age of 65 were hit (9/100,000).

A national vaccination campaign aiming at vaccinating the whole population above 6 months of age was launched in October 2009. Pandemrix® (GlaxoSmithKline, Rixensart, Belgium), a monovalent, inactivated, AS03-adjuvanted vaccine was used. Vaccinations started in week 42, when the vaccine became available. When the campaign ended in March 2010 60% of the population had received at least one dose of the vaccine. There was no national registration of the vaccinations, but some counties kept registers of the vaccinated individuals. In this study age aggregated vaccination data from Stockholm county was used for comparison with the seroepidemiological data.

In 2010–2011, a new wave of pandemic A(H1N1)pdm09 reached Sweden. The disease was still notifiable and 1129 laboratory-confirmed cases were reported. The death toll was low, 1.1/10^6^ population (http://smi.se/upload/Publikationer/Influensa-in-Sweden-2010-2011_2011-15-3.pdf), in comparison with many other western European countries.

Standardised hemagglutination inhibition (HI) tests have been the norm for evaluation of protection against influenza and vaccine match to the epidemic strain for decades [2]. Although protection against influenza disease is multifactorial, involving both innate, adaptive, humoral and cellular immune responses and targets multiple viral antigens [3], a correlation between strain-specific serum IgG HI titres and protection against influenza infection has been identified [4]. The exact contribution of various antibody or cellular immune responses to protection is not known and most assays other than HI are not standardized. Therefore, HI tests are currently the only option for evaluation of exposure and protection.

We performed a seroepidemiological study in order to evaluate the magnitude of early spread of the infection during the summer and autumn 2009, and the long-term post-pandemic and post-vaccination prevalence of protective antibodies in various age-groups. Serum samples representative of the Swedish population were collected at four time points: in 2007, before the pandemic, in October 2009, just before the major peak and the vaccination campaign, in May 2010, approximately five months after the end of the epidemic and one year later in May 2011. The sera were examined for HI activity against HA of the A/California/7/2009 (H1N1) strain.

## Materials and Methods

### Ethics Statement

The Swedish Institute for Infectious Disease Control hereby certifies that ethical permission and use of informed consent was not required prior to collection and study of the samples in question. The reason for this conclusion is the fact that these samples cannot be traced back and connected to any individual. The Swedish Ethical Review Act (2003∶460), Ethical Review of Research Involving Humans, is therefore not applicable (see sections 1–4, http://www.epn.se/media/45159/the_etical_review_act.pdf). There is no other legislation in force in Sweden that alters this conclusion.

### Serum Specimens

Serum samples from 2007 (collected: 2058, analysed: n = 1968) were obtained from a cross-sectional study for follow-up of **t**he Swedish national vaccination programme [5]. The samples were collected from individuals in the Swedish population register using a randomized sample stratified for age groups. Sera from October 2009, before the vaccination campaign and the pandemic outbreak (collected: n = 2220, analysed: n = 2218), May 2010, approximately five months after the end of the pandemic outbreak and the vaccination campaign (collected: n = 2663, analysed: n = 2638), and May 2011 (collected: n = 2521, analysed: n = 2513) were anonymised leftover diagnostic samples from six (2009), eight (2010) or nine (2011) clinical chemistry laboratories across the country. Samples were optimally allocated over age and geographical strata to minimise the variance of the estimates. Population data were obtained from Statistics Sweden (www.scb.se). The number of serum samples analysed in each age group is shown in Table 1. Age-groups are given as e.g. “0–1 years”, where the last digit represents “being one but not two years old”.

**Table 1:** Number of tested serum samples per age-group and collection period (percent of total).

Age group (years)	Sample collection period			
	2007	October 2009	May 2010	May 2011
0–1	22 (1.1%)	80 (3.6%)	93 (3.5%)	72 (2.9%)
2–4	113 (5.7%)	65 (2.9%)	106 (4.0%)	82 (3.3%)
5–14	608 (30.9%)	239 (10.8%)	282 (10.7%)	271 (10.8%)
15–24	340 (17.3%)	319 (14.4%)	340 (12.9%)	324 (12.9%)
25–35	87 (4.4%)	420 (18.9%)	358 (13.6%)	357 (14.2%)
36–49	152 (7.7%)	316 (14.2%)	485 (18.4%)	472 (18.8%)
50–64	253 (12.9%)	445 (20.1%)	503 (19.1%)	484 (19.3%)
65–74	222 (11.3%)	165 (7.4%)	238 (9.0%)	237 (9.4%)
75–79	64 (3.3%)	70 (3.2%)	93 (3.5%)	81 (3.2%)
≥80	107 (5.4%)	99 (4.5%)	140 (5.3%)	133 (5.3%)
**All ages**	**1968**	**2218**	**2638**	**2513**

### Pandemic Vaccine Uptake and Laboratory Influenza Surveillance

The mode of registration of vaccinations varied between the 21 counties in Sweden, and aggregated data were sent weekly to the Swedish Institute for Communicable Disease Control. Some counties, among those Stockholm county with around 2,000,000 inhabitants, kept a vaccination register with the 12-digit Swedish personal number as unique identifier and could provide age-stratified data. All laboratory-verified influenza diagnoses in Sweden were reported with the personal numbers in the web-based reporting system, SmiNet, according to the law. The personal number is based on date of birth, and also gives information about the sex of the patient.

### Hemagglutination Inhibition (HI) Test

Serial two-fold dilutions of the sera, from 1/10 to 1/640, were tested in a standard HI assay [6] using chicken red blood cells. All sera were treated with receptor destroying enzyme before testing. The virus antigen NYMC X-179A (GSK Biologicals, Dresden, Germany), was the same strain that was used in the pandemic vaccine, a laboratory reassortant with the HA gene donated from A/California/7/2009 (H1N1)v, and was not inactivated. The assay was semi-automated using a Hamilton Microlab STARlet liquid handling workstation (Hamilton Robotics GmbH, Martinsried, Germany). Titres (the reciprocal of the highest serum dilution giving a positive result) ≥40 were considered 50% protective [7], [8] and titres ≥10 as seropositive. An in-house internal control serum was included in each plate, and an international standard (09/194, NIBSC, Potters Bar, UK) was included in each assay. A deviation of more than one titre step from the predetermined titre of any of the controls resulted in retesting of the samples.

### Statistical Methods

Percentages of serum samples with HI titres ≥10 and ≥40 in the different age groups as well as estimates for the national level are presented with 95% exact confidence intervals. The national estimates are age-adjusted according to the Swedish population structure. Differences in percentages of serum samples with HI titres ≥10 and ≥40 within age groups between 2010 and 2011 were tested with chi square test. Median HI titres for seropositive individuals (HI titre ≥10) in the different age groups were calculated for 2010 and 2011 and tested with Mann Witney U test. The statistical software R version 2.9.2 was used for statistical analysis.

## Results

### Pre- and Post-pandemic HI Antibodies

**Figure 1:**
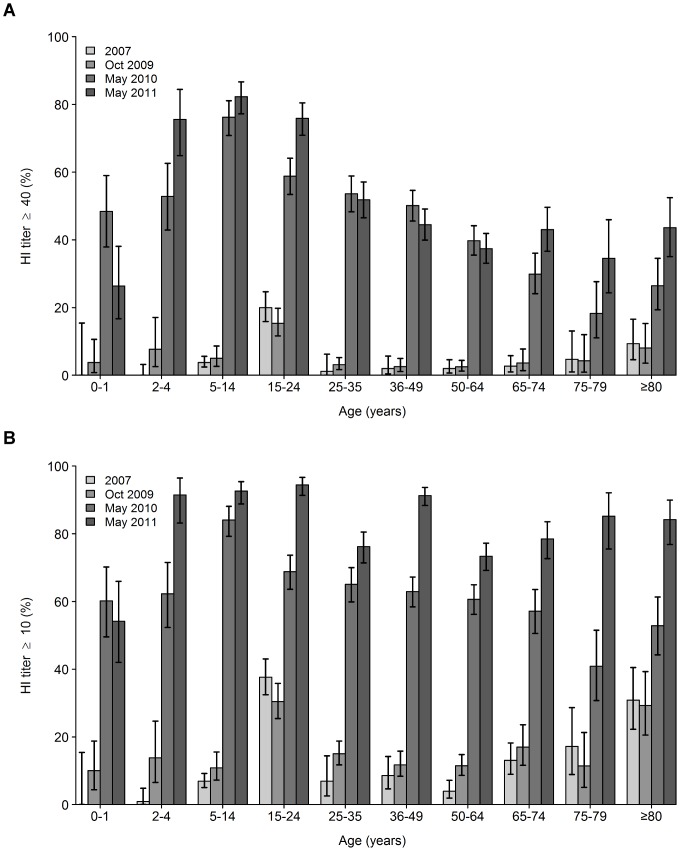
Prevalence of HI titers ≥40 and ≥10 against influenza A(H1N1)pdm09. Percentage of individuals with HI titers (A) ≥40 and (B) ≥10 against influenza A(H1N1)pdm09 in serum samples collected in 2007, October 2009, May 2010 and May 2011. Bars indicate 95% confidence intervals.

To investigate the pre-pandemic level of A(H1N1)pdm09 HI reactivity in Sweden 1968 serum samples collected in 2007 were analysed. The population prevalance of HI titres ≥40 was 4.9%; highest was the age group 15–24 (20.0%) followed by ≥80 year-olds (9.3%; Fig. 1A). The population prevalence of detectable antibody (HI titre ≥10) was 12.5% (Fig. 1B).

New serum samples (n = 2218) were collected in October 2009, just before massive spread of the virus had begun in Sweden and before the national vaccination campaign. The population prevalence of HI titres ≥40 was 5.4%; highest was the age group 15–24 years (15.4%; Fig. 1A). The prevalence in persons ≥80 years was 8.1%. The population prevalence of detectable antibody was 16.4%, with similar figures in persons 15–24 years (30.4%) and ≥80 years (29.3%; Fig. 1B).

**Figure 2:**
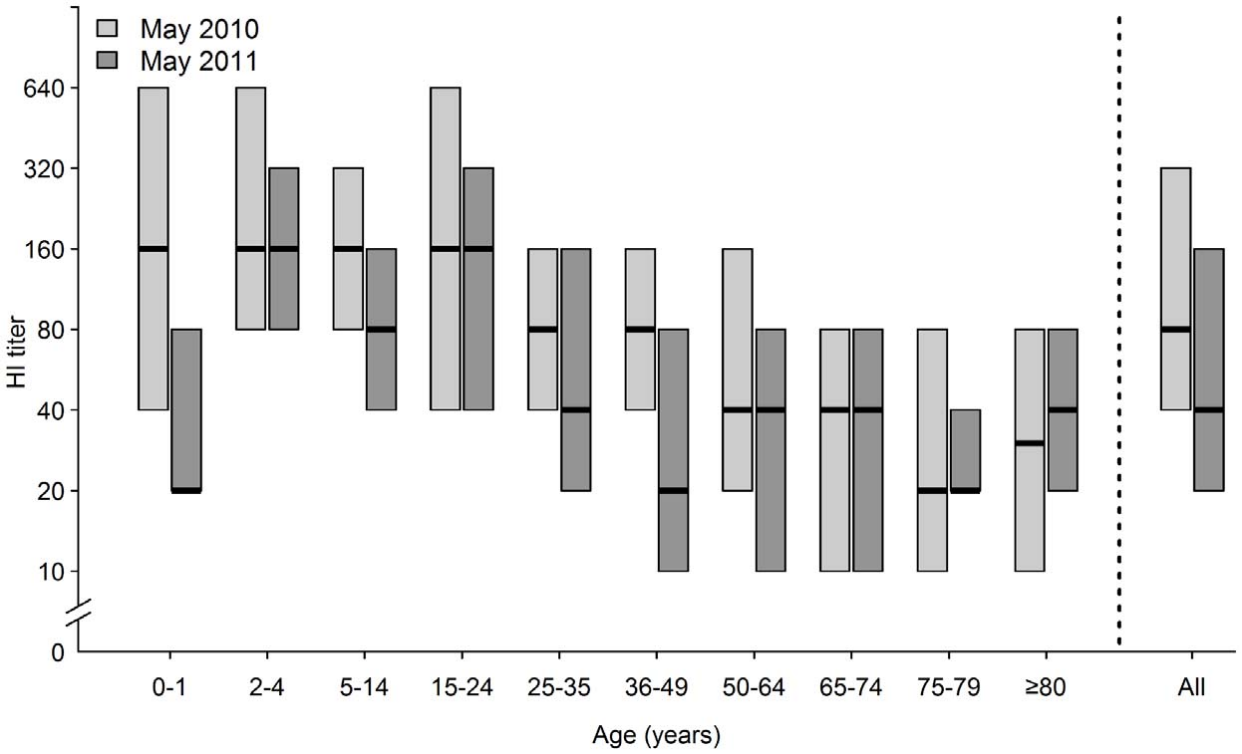
HI titers against influenza A(H1N1)pdm09. HI titers against influenza A(H1N1)pdm09 in seropositive individuals (HI ≥10) in May 2010 and May 2011. Horizontal bars show medians, while boxes show 25th and 75th percentiles. Sera were not titrated further than 640.

In May 2010, approximately five months after the end of the epidemic and the vaccination campaign, 2638 serum samples were collected and analysed. The prevalence of HI titres ≥40 had increased significantly in all age-groups since October 2009. The population prevalence was 48.6%. It was highest in children 5–14 years of age (76.2%) and declined with lower or higher age (48.4% for 0–1 year-olds and lowest, 18.3%, for 75–79 year-olds; Fig. 1A). The population prevalence of detectable antibody was 64.0% (Fig. 1B). The prevalence of detectable antibody did not decline with increasing age to the same extent as the HI titre ≥40 prevalence. The median HI titre in seropositive individuals (HI titre ≥10) was 80, reaching 160 in persons ≤24 years, and declining with increasing age to 20 in 75–79 year-olds (Fig. 2).

One year later, in May 2011, a new serum panel was collected, and 2513 serum samples were analysed. The population prevalence of HI titres ≥40 was 52.2%, a statistically significant increase compared to 2010 (p = 0.005). Increases compared to 2010 were seen in 2–4 year-olds (75.6% vs. 52.8%, p = 0.002), 15–24 year-olds (75.9% vs. 58.8%, p<0.0001), 65–74 year-olds (43.0% vs. 29.8%, p = 0.004), 75–79 year-olds (34.6% vs. 18.3%, p = 0.02) and ≥80 year-olds (43.6% vs. 26.4%, p = 0.004; Fig. 1A). In the 0–1 year-olds, in this serum panel born after the pandemic outbreak and the vaccination campaign, the prevalence was significantly lower than in the same age-group in 2010 (26.4% vs. 48.4%, p = 0.007). The population prevalence of detectable antibody was 83.8%, a substantial increase compared to 2010 (p<0.0001). The prevalence of detectable antibody increased significantly in all age-groups, except in the 0–1 year-olds (Fig. 1B; p-values not shown).

The median HI titer in the population decreased from 80 to 40 between May 2010 and May 2011 (p<0.001; Fig. 2), with significant decreases in the age-groups 0–1, 5–14, 15–24, 25–35, 36–49 and 50–64 years. The largest decrease was in 36–49 year-olds, where the median titer decreased from 80 to 20.

### Post-pandemic Seroprevalence in Relation to Pandemic Vaccine Uptake and the 2009–2010 and 2010–2011 Seasons A(H1N1)pdm09 Infection Incidence

**Figure 3:**
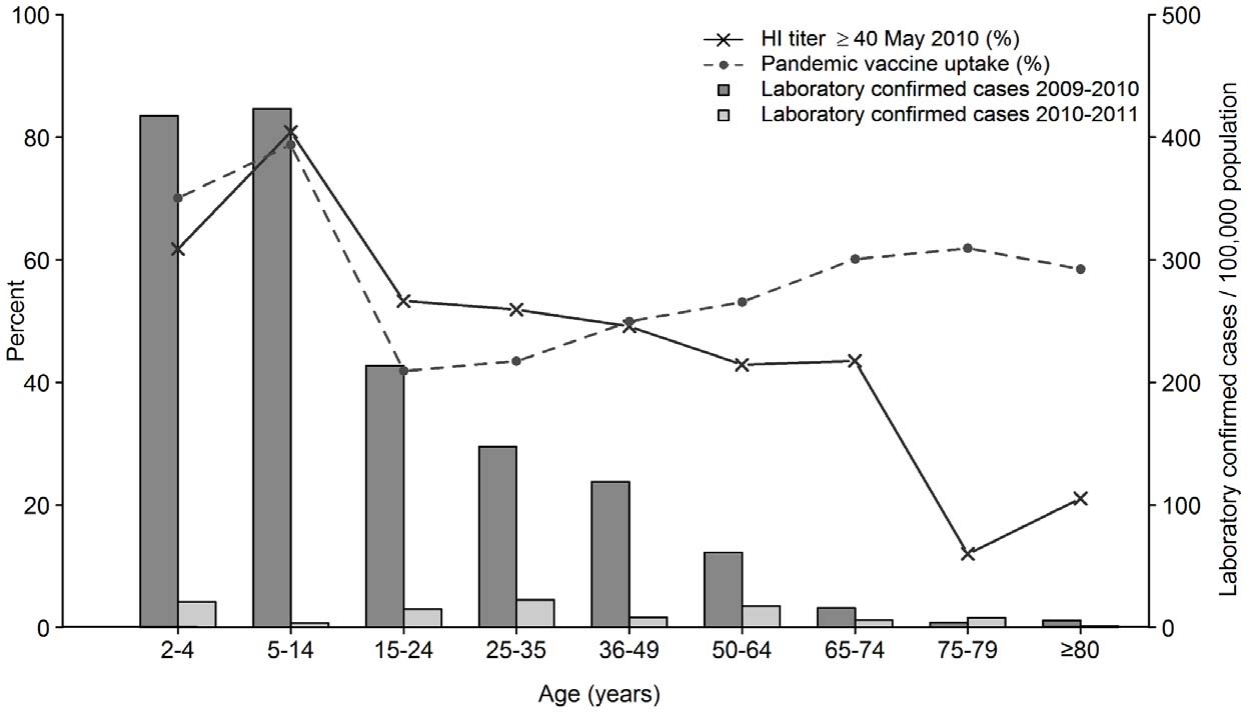
Prevalence of HI titers ≥40, pandemic vaccine uptake and laboratory-confirmed cases in Stockholm county. Influenza A(H1N1)pdm09 in Stockholm county the 2009–2010 and 2010–2011 seasons. Post-pandemic prevalence of HI titers ≥40 (samples collected in May 2010), pandemic vaccine uptake and laboratory-confirmed cases for the 2009–2010 and 2010–2011 seasons.

Figure 3 shows post-pandemic seroprevalence (May 2010) in relation to vaccine uptake and incidence of infection. As there was no complete vaccination register for the whole country Stockholm county, encompassing around 20% of the Swedish population, was chosen as an example. Eighthundred-and-fifty-one of the 2638 samples collected in May 2010 were obtained from this county. Since the serum samples were anonymised, they could not be connected to vaccination or infection on an individual level.

The total pandemic vaccine uptake in Stockholm was 54%, slightly lower than in the whole country (60%). The uptake was highest in 5–14 year-olds and lowest in 15–24 year-olds (Fig. 3). The 75–79 year-olds had the highest vaccine uptake among adults. The post-pandemic seroprevalence in May 2010 shown i Fig. 3 resembled that of the whole country.

The incidence of reported laboratory-confirmed A(H1N1)2009A(H1N1)pdm09 infections per 100,000 population in Stockholm during the 2009–2010 season was highest in children, from 383 in 0–1 year olds to 423 in 5–14 year olds (Fig. 3). The incidence then declined with increasing age to only 4 in 75–79 year-olds. The overall incidence was 166/100,000.

The 2010–2011 season epidemic of A(H1N1)pdm09 gave a much lower incidence (Fig. 3). In Stockholm county the incidence of laboratory-verified infections from week 40 in 2010 to week 20 in 2011 was 14,8 per 100,000 population. While in 2009–2010 the highest incidence was found in 5–14 year-olds, this age group had very low incidence, 3.5/100,000, in the 2010–2011 season.

## Discussion and Conclusion

A low prevalence of HI antibodies to the pandemic A(H1N1)pdm09 virus was detectable in the Swedish population before the pandemic, with the highest seroprevalence found in young adults. Infection and mass-vaccination during the pandemic 2009–2010 resulted in high and long-lasting seroprevalence, especially in children of school age. Persons above 65 years were an exception; despite high vaccine coverage only between 20% and 30% had HI titres ≥40 in May 2010. However, an increase was observed in 2011, possibly related to boosting by seasonal vaccination, which was estimated to cover more than 50% of the persons >65 years in the fall 2010. The seroprevalence was still below that in other age-groups, but despite this they had the lowest incidence of disease, both during the pandemic and during the first post-pandemic wave in 2010–2011.

Most of the pre-pandemic seroprevalence observed in the 2007 and 2009 serum panels likely reflects cross-reactive antibodies elicited by previous seasonal H1N1 infections. The reason for the relatively high seroprevalence in the 15–24 year-olds before the pandemic is unknown. Elevated levels in similar age strata were reported also from Norway [9], the UK [10], Germany [11] and Greece [12], but not from Finland [13], USA [14], [15] or Japan [16]. Some countries reported relatively high pre-pandemic seroprevalence in persons >60 years, e.g. the UK [10], Greece [12] and USA [14], [15], but this was not found in Sweden. Pre-pandemic HI titres of ≥40 were however observed in 9% of ≥80 year-olds. This may be explained by the fact that persons born in 1921 or earlier (88 years or older in 2009) are likely to have experienced the Spanish flu, which was antigenically similar to A(H1N1)pdm09 [13].

The lack of over-all increase in seroprevalence between 2007 and October 2009 in Sweden indicates that no large “silent” A(H1N1)pdm09 epidemic was present before the intense spread of the virus and the national vaccination campaign that started in mid-October 2009. The appearance in October 2009 of HI antibodies in children up to four years of age may be a result of the minor peaks of H1N1-activity during the summer 2009 [1]. However, the increase in reported cases during this period affected all ages up to 40 years. It is possible that many children had less severe symptoms than the older population and were therefore not sampled to the same extent, leading to an underestimation of cases in this age group. The different sampling methods used in the 2007 and 2009 serosurveys may also influence the seroprevalence comparison at these two time points.

The post-pandemic, post-vaccination seroprevalence was clearly highest in the 5–14 year-olds, likely reflecting a combination of the high vaccine coverage and the high incidence of infection in this age group. However, in the older age groups, in particular individuals ≥75 years, the post-pandemic, post-vaccination seroprevalence was remarkably low, despite a high vaccine uptake. The median titres among seropositives in these age groups were also low. Previous studies have also observed poor antibody responses to influenza vaccination in elderly (reviewed in [17]). There are several possible explanations for this, such as immune senescence or age related diseases [18].

Considering their low prevalence of HI antibodies before the pandemic and their very low incidence of infection, the elderly must have been protected against disease by mechanisms other than blocking of viral receptor binding. This is in agreement with the only published randomized placebo-controlled influenza vaccine study in elderly [19], [20], in which protection was not correlated to HI antibody responses. Recent studies with new methodology have demonstrated antibody responses to the A(H1N1)pdm09 HA-protein with broader epitope repertoire and higher avidity in elderly compared to young individuals. These differences were not revealed in the HI test [21], [22]. Thus, it is possible that vaccination induced HA-antibodies in the elderly that were protective but not detected in the HI test.

The increased seroprevalence between May 2010 and May 2011 should have been caused by exposure to the HA protein of A(H1N1)pdm09 either through infection or vaccination. However, it should be noted that neither vaccination nor infection induce antibodies in all individuals [23], and an estimate of true disease incidence or vaccination uptake cannot be made from serologic studies. For HI titre ≥40 significant increases in prevalence were observed among 2–4 and 15–24 year-olds as well as in the above mentioned age-groups above 65 years. Apart from for the elderly, vaccination is not a likely explanation for this since children and young adults are rarely vaccinated against seasonal influenza in Sweden. The increases in these age groups should therefore be caused by A(H1N1)pdm09 infection rather than by vaccination. Indeed, the number of laboratory-confirmed cases in these two age groups in 2010–2011 was approximately three times higher than that in 5–14 year-olds (15 vs. 5 cases/100,000). The 5–14 year-olds had the highest prevalence of HI titers ≥40 and were thus probably better protected against infection. Although the numbers of laboratory-confirmed cases are low they may reflect a wider spread of mild and therefore unnoticed A(H1N1)pdm09 infection that could explain the observed increases in seroprevalence.

The over-all rise in prevalence for detectable antibody (HI titre ≥10) was greater than for titre ≥40, with significant increases in all age-groups except 0–1 year-olds. The discrepancy was most striking in people above 36 years, perhaps reflecting an aging immune system with a broad repertoire but decreased ability to mount responses of high magnitude. Increasing A(H1N1)pdm09 seroprevalence between 2010 and 2011 has also been reported from other countries [24]–[26]. Despite the over-all increase in seroprevalence between May 2010 and May 2011 there was an over-all decrease in median HI titer among responders. This is not as contradictory as it may seem, as the major seroprevalence increase was in the ≥10–40 HI titre interval.

The proportions of post-pandemic seropositives resulting from infection vs. vaccination in 2009 are not known. A large part of the population had not yet been vaccinated, or had not yet developed antibodies from recent vaccination, when the peak of the epidemic occurred. However, considering a vaccine uptake of 60% in the population and the high immunogenicity of Pandemrix® [27], [28], with HI titres ≥40 sustained in 70% of healthy adult vaccinees after 6 months (unpublished data), we assume that vaccination should have contributed to the high seroprevalence in May 2010. An indirect proof of the vaccine contribution to immunity is the development of the A(H1N1)pdm09 epidemic in 2010–2011. Although direct country comparisons are hampered by the different mortality- and severe disease-data collection methods it seems that Sweden was far less severely hit concerning severe H1N1 illness than many other countries in Western Europe. By the end of April 2011, 10 deaths (1.1/10^6^ population) and 63 (6.6/10^6^)) patients admitted to intensive care had been reported to SMI for the 2010–2011 season (http://smi.se/upload/Publikationer/Influensa-in-Sweden-2010-2011_2011-15-3.pdf). In e.g England the death toll was reported to be 9.2/10^6^, while the cumulative number of critical care beds occupied with suspected or confirmed influenza cases was 200–437/10^6^ (depending on the region).

A limitation of the present study is that while the 2007 serum samples were collected based on a random sampling design [5], the 2009, 2010 and 2011 samples were instead collected using convenience sampling. Therefore, in 2009, 2010 and 2011, only persons seeking medical care, and having blood samples taken for clinical chemistry (note: not for microbiological) laboratory analysis, were included. We cannot exclude the possibility of a selection bias for the 2009, 2010 and 2011 samples, as persons seeking medical care could have an increased or decreased influenza morbidity, and perhaps influenza vaccine coverage, compared with the general population. Individuals belonging to an influenza risk group may be overrepresented among persons seeking medical care, but they do not have an increased risk of infection. It could be speculated, however, that they have an increased likelihood of previously having been vaccinated against influenza. Despite these limitations, the employed collection method has been used in similar seropidemiological studies [9], [10], [25] and was the only feasible option for rapid serum sampling before and after the pandemic.

In summary, this seroepidemiological study on samples collected in Sweden before and after the 2009–2010 influenza pandemic showed low prevalence of pre-existing A(H1N1)pdm09 HI-antibody in the population, while natural infections and the vaccination campaign during the pandemic elicited high and long-lasting antibody responses, especially in children. The elderly appear to have been largely protected against disease despite low pre-pandemic antibody prevalence and poor HI-antibody response to the vaccine. Better understanding of the correlates of protection and use of alternative laboratory test methods will be needed to better predict spread of and vulnerability to future pandemic influenza viruses.
